# Serum proton NMR metabolomics analysis of human lung cancer following microwave ablation

**DOI:** 10.1186/s13014-018-0982-5

**Published:** 2018-03-12

**Authors:** Jian-Ming Hu, Huang-Tao Sun

**Affiliations:** Thoracic surgeons, Jiangxi Provincial People’s Hospital, 152 Patriotic Road, Nanchang City, 333000 People’s Republic of China

**Keywords:** Non-small cell lung cancer, Microwave ablation, Nuclear magnetic resonance, Metabolomics, PLS-DA

## Abstract

**Background:**

To find potential serum biomarkers of microwave ablation (MWA) for treatment of human lung cancer by ^1^H nuclear magnetic resonance (NMR)-based metabolomics analysis.

**Methods:**

Serum specimens collected from 43 healthy individuals, 39 patients with advanced non-small cell lung cancer (NSCLC) and 38 NSCLC patients treated with MWA, were subjected to ^1^H NMR-based metabolomics analysis. Partial least squares discriminant analysis was used to analyze the data.

**Results:**

Compared with healthy controls, NSCLC patients showed significantly elevated serum levels of lactate, alanine, glutamate, proline, glycoprotein, phenylalanine, tyrosine and tryptophan, and markedly decreased serum levels of glucose, taurine, glutamine, glycine, phosphocreatine and threonine (*p* < 0.05). MWA treatment reversed the metabolic profiles of NSCLC patients towards the control group.

**Conclusions:**

^1^H NMR-based metabolomics analysis enhanced the current understanding of the mechanisms involved in NSCLC, and uncovered the therapeutic potential of MWA for treatment of NSCLC. The above disturbed serum metabolites were proposed to be the potential biomarkers that may help to predict NSCLC and to evaluate the efficacy of MWA in the treatment of NSCLC.

**Electronic supplementary material:**

The online version of this article (10.1186/s13014-018-0982-5) contains supplementary material, which is available to authorized users.

## Background

In China, lung cancer is the most common incident cancer and the leading cause of cancer death for both men and women in 2015 [1]. It was reported that 486,555 patients died from lung cancer and the 5 year survival rate for patients was less than 20% in 2010 [2]. Surgery is the most preferred and effective method for lung cancer patients. However, many patients are inoperable such as advanced non-small cell lung cancer patients and lung metastases patients. At present, minimally invasive thermal therapy including radiofrequency ablation (RFA) and microwave ablation (MWA) are widely used in lung cancer patients in clinic [3], which use heat generated by the biological effects of tumor cells directly to cause irreversible damage or coagulation necrosis in tumor tissues [4].

MWA has several advantages such better heating of larger tumour volumes, which is consider to be more suitable for lung tissues [5]. Therapeutic effect evaluation of MWA is very important for doctors to easily and timely adjust the treatment plan to maximize the efficacy of thermal ablation therapy. Efficacy evaluation of thermal ablation is currently based on the anatomical imaging, morphology or pathology examination. The major disadvantages of these traditional indicators include poor sensitivity and specificity, which cannot effectively estimate the curative effects and predict prognosis of NSCLC patients [6].

A number of NMR-based metabolomics studies have reported biomarkers that effectively discriminate between NSCLC subjects and healthy controls [7–9]. Deja and co-workers proposed that the the following metabolite biomarkers could potentially be useful in distinguishing lung cancer states: isoleucine, acetoacetate, and creatine as well as the two NMR signals of N-acetylated glycoproteins and glycerol [10]. Rocha investigated the metabolic profile variations of plasma from lung cancer patients and healthy controls through NMR-based metabolomics. Relatively lower high density lipoprotein (HDL) and higher very low density lipoprotein (VLDL) and low-density lipoprotein (LDL) in the patients’ plasma, together with increased lactate and pyruvate and decreased levels of glucose, citrate, formate, acetate, several amino acids and methanol, were detected [11]. In another study of temporal characterization of serum metabolite signatures in lung cancer patients undergoing chemotherapy ± radiation treatment, the feasibility of metabolites in the plasma of lung cancer patients as temporal biomarkers of clinical outcomes were discussed [12]. However, few studies take advantage of NMR spectroscopy’s ability to obtain a metabolic fingerprint of NSCLC patients after MWA treatment.

To further investigate the overall metabolic profiling of NSCLC patients and the efficacy of microwave thermal ablation in the treatment of NSCLC, metabolomics approach was introduced to give a holistic view of endogenous metabolites of the patients, which could deepen our knowledge about non-small cell lung cancer and promote the thermal ablation treatment for lung cancer, and these should be benefit for human health in the future.

## Methods

### Ethics statement

The study was approved by the Institutional Ethics Committee of the Jiangxi Provincial People’s Hospital. A written informed consent was obtained from all participants involved in this study.

### Patients and samples

Between 20 February 2014 and 30 May 2016 patients who met the following criteria were retrospectively enrolled in the study: (1) pathologically verified peripheral NSCLC, (2) stage IIIB or IV, (3) chemotherapy-naive (except patients with recurrence treated with adjuvant chemotherapy or adjuvant radiation), (4) an Eastern Cooperation Oncology Group (ECOG) performance status of 0 to 2, and (5) adequate pulmonary, cardiac, hepatic, renal and hematological functions to allow anticancer treatment.

Selected characteristics of the NSCLC cases and controls were summarized in Table 1. Blood samples were collected prior to the morning meal, and serums were obtained by centrifugation of the blood samples at 3500 rpm for 10 min at 4 °C. Aliquots of the serum samples were stored at − 80 °C until NMR analysis.

**Table 1:** Clinicopathological characteristics of the enrolled NSCLC patients, MWA treated patients and healthy controls

Characteristic	NSCLC	MWA	Healthy
Number	38	39	43
Gender			
Male	19	20	22
Female	19	19	21
Age (years)			
≥ 60	21	20	21
< 60	17	19	22
Stage			
IIIB	10	13	–
IV	28	26	–
Tumour location			
Peripheral	38	39	–
Central	0	0	–
Tumour size (cm)			
≥ 3.5	21	22	–
< 3.5	17	17	–
ECOG performance status			
0	0	2	–
1	36	35	–
2	2	2	–
Pathology			
Adenocarcinoma	19	20	–
Squamous carcinoma	13	10	–
Adenosquamous carcinoma	6	8	–
Large cell carcinoma	0	1	–
MWA time (min), mean (range)	–	14.0 (4.0–42.0)	–
MWA power			
70	–	33	–
60	–	6	–
Number of antennas			
One	–	16	–
Two	–	23	–

### Serum preparation and ^1^H NMR spectroscopy

About 1 mL serum samples were deproteinized by methanol with the ratios of serum: methanol as 1: 2 (*v*/v). The mixtures were vortexed and incubated at − 20 °C for about 30 min, and then were centrifuged into pellet proteins at 12000 g for 30 min. The supernatants were transferred into fresh tubes and lyophilized. The dried samples were dissolved in 600 μL 99.8% D_2_O phosphate buffer (0.2 M, pH at 7.0) containing 0.05% sodium salt of 3-trimethylsilylpropionic acid (TSP, *w*/*v*), vortexed, centrifuged and decanted to 5 mm NMR tubes.

All ^1^H NMR spectra were recorded with a Bruker AV 500 MHz spectrometer. A transverse relaxation-edited Carr-Purcell-Meiboom-Gill sequence [recycle delay-90-(τ-180-τ)n-acquisition] with a total spin echo delay (2nτ) of 40 ms was used to attenuate broad signals from slowly tumbling molecules such as proteins, whereupon the signals of the micro-molecule metabolites were clearly observed. ^1^H NMR spectra were measured with 128 scans into 64 K data points with a recycle delay of 3 s over a spectral width of 20 ppm. The spectra were Fourier-transformed after multiplication by an exponential window function corresponding to a line broadening of 0.5 Hz. Resonances were assigned by querying the database HMDB (http://www.hmdb.ca/), MMCD (http://mmcd.nmrfam.wisc.edu/), and were aided by the Chenomx NMR suite (version 8.0, Chenomx, Inc.).

### Data processing and multivariate analysis

The ^1^H NMR spectra were manually phased and baseline-corrected by MestReNova (version 8.0.1,Mestrelab Research SL). Spectra were aligned to the TSP signal at zero ppm. A linear interpolation method was used to align the spectra using MestReNova. Regions containing residual water and methanol signals were removed, and the spectra were binned into integrated segments with equal widths of 0.01 ppm and adjusted by probabilistic quotient normalization and pareto-scaling prior to multivariate analysis.

First, an unsupervised principal component analysis (PCA) was used for for the metabolomics data overview and the spotting of outliers, and then for the detection of any grouping. Clustering was failed for the dataset (Additional file 1: Figure S1). Then a supervised partial least squares discriminant analysis (PLS-DA) was used to gain valuable insights on group-predictive spectral features. The metabolic profiles could be visualized as score plot, where each point represents a sample. The corresponding loading plot and S-plot were generated to provide information on the metabolites that influence clustering of the samples. In addition, a correlation circle plot produced by sparse PLS regression was applied to illustrate the relationships between certain integral metabolites and groupings.

To quantitatively assess the performance of the model, a repeated two fold cross-validation and permutation testing was carried out. Permutation distribution of the test statistic was computed based on 10,000 times repeated random permutation of the class labels and the significance value was taken to be the fraction of samples not exceeding the test statistic for the original sample. The overall quality of the model was evaluated by the cumulative R^2^, while the predictive ability was assessed by the cumulative Q^2^. Integration areas of the detected metabolites with marked differentiating ability were first tested for distribution normality. The student t-test or rank test was then employed to detect differences in metabolite levels between groups and *p* < 0.05 was considered to be statistically significant.

### Pathway analysis

Differential metabolites were subjected to pathway analysis by MetaboAnalyst [13], which combines results from powerful pathway enrichment analysis with the pathway topology analysis, to identify the most relevant pathways involved in the NSCLC patients.

In order to excavate anything gene as potential biomarkers, and to integrate gene and metabolomics information to provide a better understanding of the efficacy of MWA in the treatment of NSCLC, the identified metabolites in metabolomics were then mapped to the KEGG pathway for biological interpretation of higher-level systemic functions. The metabolites and corresponding pathways were visualized using KEGG Mapper tool (http://www.genome.jp/kegg/mapper.html).

## Results

### ^1^H NMR spectra

**Fig. 1:**
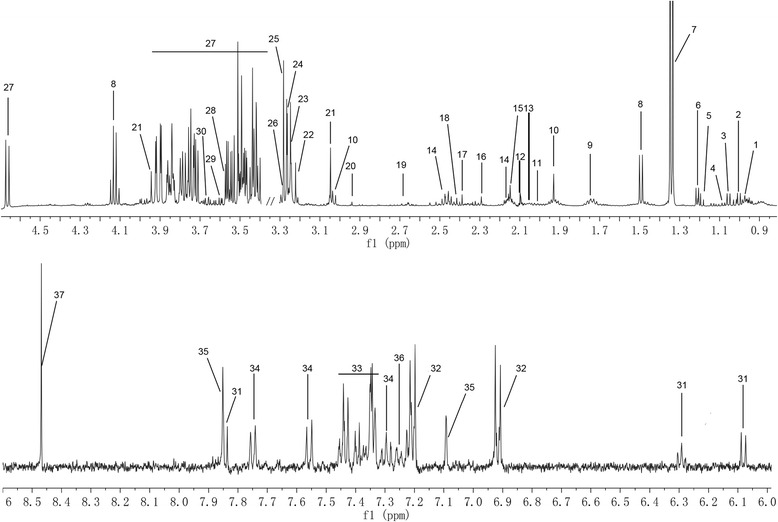
Representative ^1^H NMR spectra of serum samples with metabolites labeled. 1 Isoleucine; 2 Leucine; 3 Valine; 4 Isobutyrate; 5 Ethanol; 6 3-Hydroxybutyrate; 7 Lactate; 8 Alanine; 9 Lysine; 10 Acetate; 11 Proline; 12 Glycoprotein; 13 Glutamate; 14 Glutamine; 15 Methionine; 16 Acetoacetate; 17 Pyruvate; 18 Succinate; 19 Aspartate; 20 Asparagine; 21 Phosphocreatine; 22 Choline; 23 O-Acetylcholine; 24 O-Phosphocholine; 25 TMAO; 26 Taurine; 27 Glucose; 28 Glycine; 29 Threonine; 30 Glycerol; 31 dCTP; 32 Tyrosine; 33 Phenylalanine; 34 Tryptophan; 35 Histidine; 36 Tyramine; 37 Formate

Representative ^1^H NMR spectra of serum samples were shown in Fig. 1, with metabolites assigned. The variations in serum metabolites between groups were summarized in Table 2. The means and standard deviation values of metabolites for each group were provided in the Additional file 1: Table S1. Compared with the control group, the NSCLC group showed elevated levels of lactate, alanine, glutamate, proline, glycoprotein, phenylalanine, tyrosine and tryptophan, and decreased levels of glucose, taurine, glutamine, glycine, phosphocreatine and threonine, which could be partially or completely reversed by MWA treatment.

**Table 2:** Potential serum biomarkers identified by ^1^H NMR and their variations among NSCLC patients, MWA treated patients and healthy controls

No.	Metabolites	Chemical shifts (ppm)	NSCLC/CTRL		MWA/NSCLC		MWA/CTRL	
			fold	*p*	fold	*p*	fold	*p*
1	Isoleucine	0.94 (t), 1.00 (d)	1.09		1.18		1.29	*
2	Leucine	0.96 (d), 0.97 (d)	1.09		0.93		1.00	
3	Valine	0.99 (d), 1.05 (d)	1.11		1.17		1.30	**
4	Isobutyrate	1.07 (d)	1.06		1.09		1.16	
5	Ethanol	1.19 (d), 3.65 (q)	1.01		1.11		1.12	
6	3-Hydroxybutyrate	1.20 (d), 2.30, 2.41 (m)	1.15		1.09		1.25	***
7	Lactate	1.33 (d), 4.12 (q)	2.13	***	0.90	*	1.92	***
8	Alanine	1.48 (d), 3.78 (q)	1.76	***	0.87	*	1.52	***
9	Lysine	1.73 (m), 1.91 (m), 3.30 (t)	1.12		1.10		1.24	***
10	Acetate	1.92 (s)	1.18		1.08		1.27	**
11	Proline	2.01 (m)	1.45	**	0.86		1.25	**
12	Glycoprotein	2.09 (s)	1.18	*	0.89		1.05	
13	Glutamate	2.05 (m), 2.12 (m), 2.36 (m)	1.14	**	0.85	***	0.96	
14	Glutamine	2.14 (m), 2.45 (m)	0.80	***	1.11		0.89	*
15	Methionine	2.14 (s), 2.65 (t)	1.10		1.11		1.22	*
16	Acetoacetate	2.28 (s)	1.15		1.19		1.37	**
17	Pyruvate	2.38 (s)	1.19		0.85		1.01	
18	Succinate	2.41 (s)	1.21		0.81		0.98	
19	Aspartate	2.68, 2.81 (m)	0.86		1.17		1.01	
20	Asparagine	2.87, 2.95 (m)	0.88		1.16		1.02	
21	Phosphocreatine	3.04 (s), 3.93 (s)	0.81	***	1.13		0.91	
22	Choline	3.20 (s)	0.89		1.07		0.95	
23	O-Acetylcholine	3.21 (s)	0.89		0.89		0.79	**
24	O-Phosphocholine	3.23 (s)	0.89		0.86		0.76	***
25	TMAO	3.27 (s)	1.07		0.89		0.95	
26	Taurine	3.27 (t), 3.42 (t)	0.76	***	1.38	***	1.05	
27	Glucose	3.4–3.92 (m)	0.92	*	1.08	*	0.99	
28	Glycine	3.56 (s)	0.89	*	1.12	*	1.00	
29	Threonine	3.59 (d), 4.25 (m)	0.82	*	1.20	**	0.98	
30	Glycerol	3.58, 3.65 (m)	1.24		0.82		1.01	
31	dCTP	6.07 (d), 6.08 (t), 7.83 (d)	0.86		0.86		0.74	***
32	Tyrosine	6.91 (d), 7.20 (d)	1.16	**	0.97		1.12	**
33	Phenylalanine	7.33 (d), 7.38 (t), 7.43 (t)	1.17	*	0.83		0.97	
34	Tryptophan	7.55 (d), 7.74 (d)	1.30	***	0.89		1.15	**
35	Histidine	7.08 (s), 7.84 (s)	0.88		1.14		1.00	
36	Tyramine	7.24 (d)	0.88		0.96		0.85	**
37	Formate	8.46 (s)	0.87		1.12		0.98	

^*1*^*H–NMR* 1H–nuclear magnetic resonance, *NSCLC* non-small-cell lung cancer, *MWA* microwave ablation, *TMAO* trimethylamine N-oxide. Asterisk P < 0.05, double asterisk *P* < 0.01 and triple asterisk *P* < 0.001. Multiplicity: s singlet, d doublet, t triplet, m multiplets

### Multivariate analysis

**Fig. 2:**
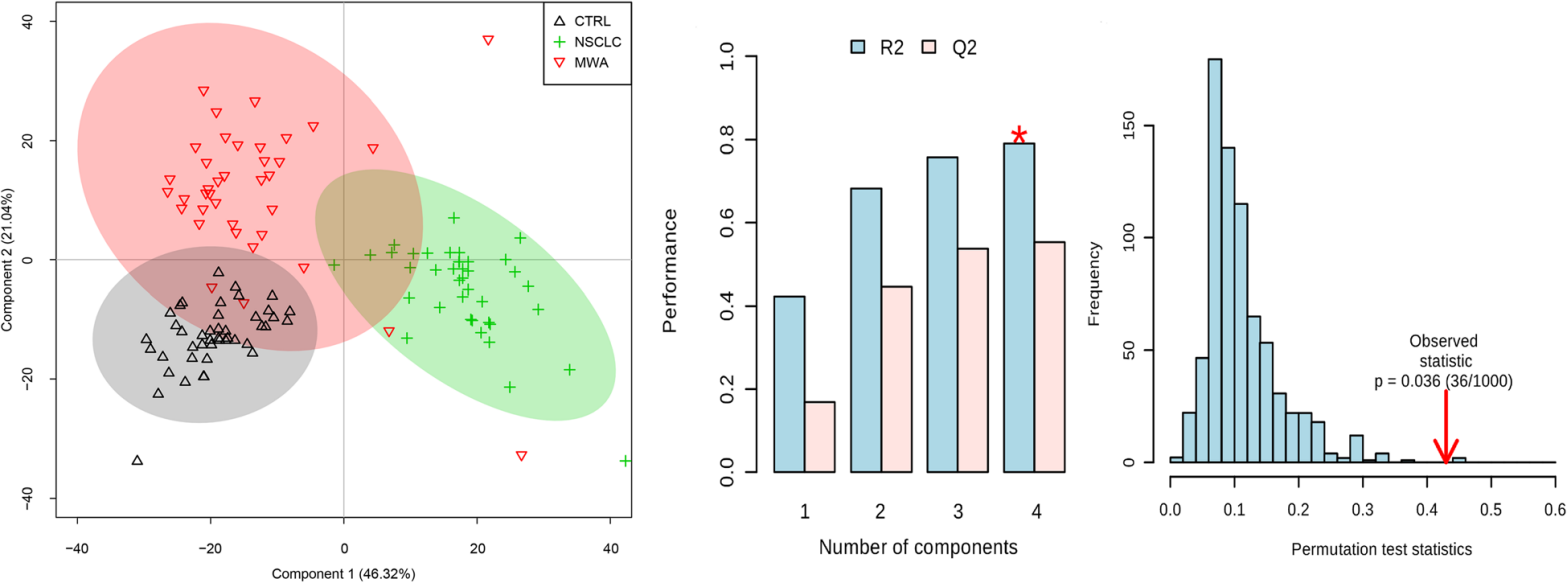
Scores plot, cross validation and a permutation test of PLS-DA model for CTRL, NSCLC and MWA groups. CTRL, healthy controls (*n* = 43). NSCLC, nonsmall-cell lung cancer patients (*n* = 38). MWA, microwave ablation treated patients (*n* = 38). The values of R^2^ (0.68) and Q^2^ (0.45) revealed satisfactory goodness of fit and goodness of prediction, respectively. The nominal *P* value (0.036) of the permutation test was less than 0.05, confirming the validity of the PLS-DA model at a 95% confidence level

**Fig. 3:**
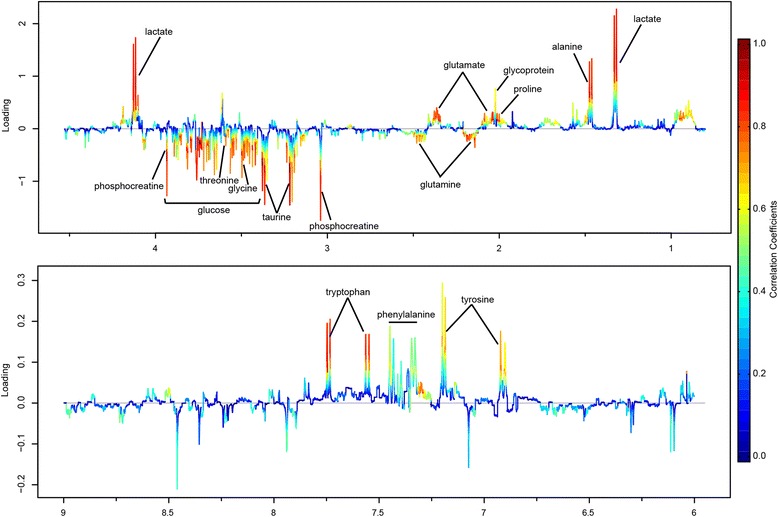
Loadings plot of PLS-DA model color-coded with the absolute value of correlation coefficients. Positive peaks corresponding to metabolites that increased in NSCLC group, and negative regions corresponding to metabolites that decreased in NSCLC group

**Fig. 4:**
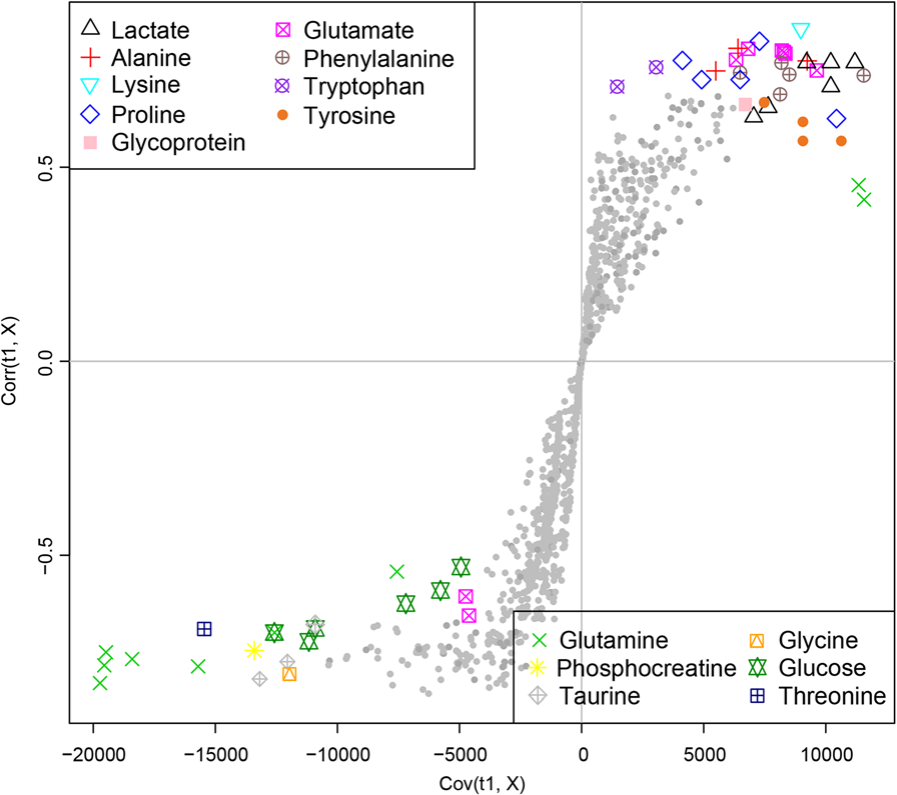
S-plot from PLS-DA analysis to visualize the variable influences and to filter potential metabolites. Warm color (red) denotes a large contribution to grouping and cold color (blue) denotes less contribution to grouping. The significantly increased metabolites in NSCLC group were located in the upper-right quadrant and the decreased metabolites were located in the lower-left quadrant

**Fig. 5:**
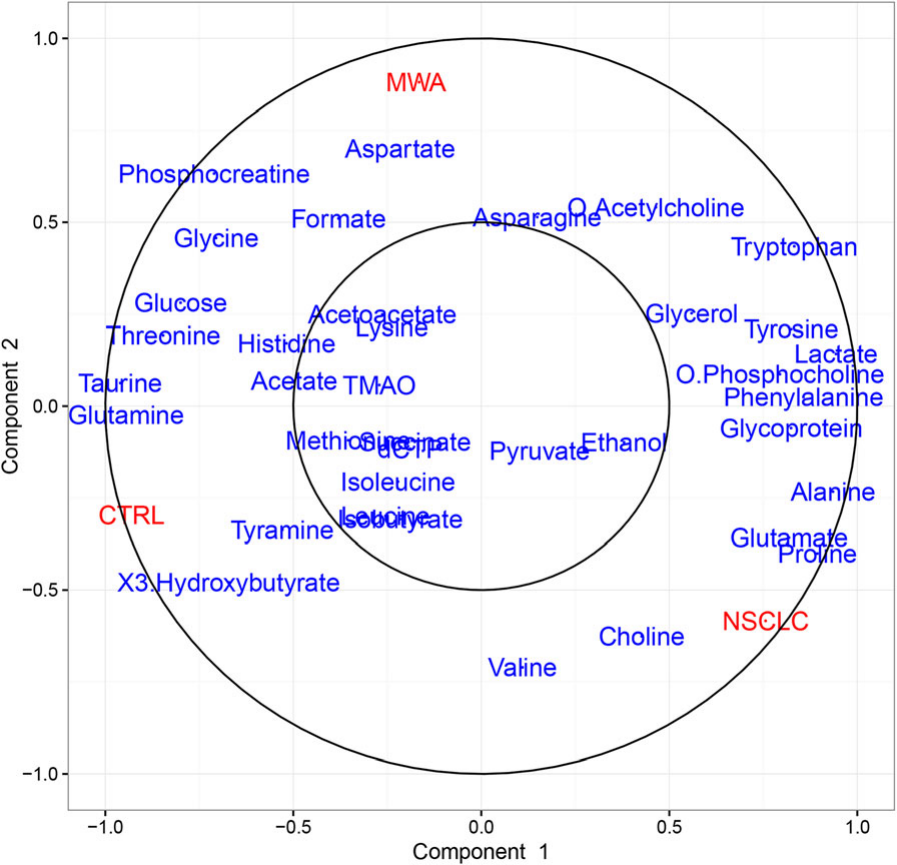
Concentric correlation circle plot concerning the correlations between the identified metabolites and the three groups, provided additional information on the endogenous metabolites among groups

The validity of the PLS-DA model against over-fitting was assessed by the parameters R^2^ (0.68), and the predictive ability was described by Q^2^ (0.45). Theoretically, the closer the R^2^ and Q^2^ value to 1, the better the PLS-DA model is. A permutation test (*n* = 1000) was then performed to assure the predictive capacity of the PLS-DA model. The observed statistic *P* values via permutation testing were 0.036 which was less than 0.05, thus confirming the validity of the PLS-DA model. The PLS-DA score plot (Fig. 2) revealed satisfactory discrimination between the three groups. MWA treatment could reverse the disturbed metabolic profile towards the control group. The corresponding color-coded coefficient loading plot on the first component (Fig. 3) and S-plot (Fig. 4) visualized the contribution of each metabolite to the separation between groups. Significantly altered metabolites between groups were the pseudo peaks in warm color in the loadings plot, and those points in the upper right and lower left quadrants of the S-plot. Peaks in the positive axis and points in the upper right quadrants means the metabolites increased in the NSCLC group. The select metabolites were graphed as scatter plots in order to show the interindividual variation (Additional file 1: Figure S2). A concentric correlation circle concerning the correlations between the identified m a status similar to healthy controls etabolites and the three groups were plotted as scatter plot, provided additional information on the endogenous metabolites among groupings (Fig. 5).

### Network visualization of metabolites and pathways

**Fig. 6:**
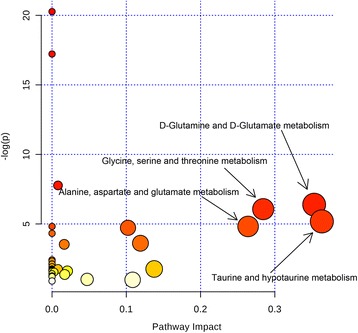
Pathway topology analysis in association with NSCLC. Bubble area donating to the impact of each pathway, with color representing the significance from highest in red to lowest in white

The evidently disturbed metabolites were subjected to pathway analysis using a web-tool MetaboAnalyst [13]. Consequently, four metabolic pathways, including taurine and hypotaurine metabolism; d-glutamine and d-glutamate metabolism; glycine, serine and threonine metabolism; alanine, aspartate and glutamate metabolism, were filtered out as the most important pathways related with the metabolic disturbances in NSCLC patients (Fig. 6). The *P* values table generated by MetaboAnalyst in association with Fig. 6 was provided in the Additional file 1: Table S2. These metabolic alterations and the associated pathways provided insights into the mechanisms involved in the development and progression of NSCLC.

**Fig. 7:**
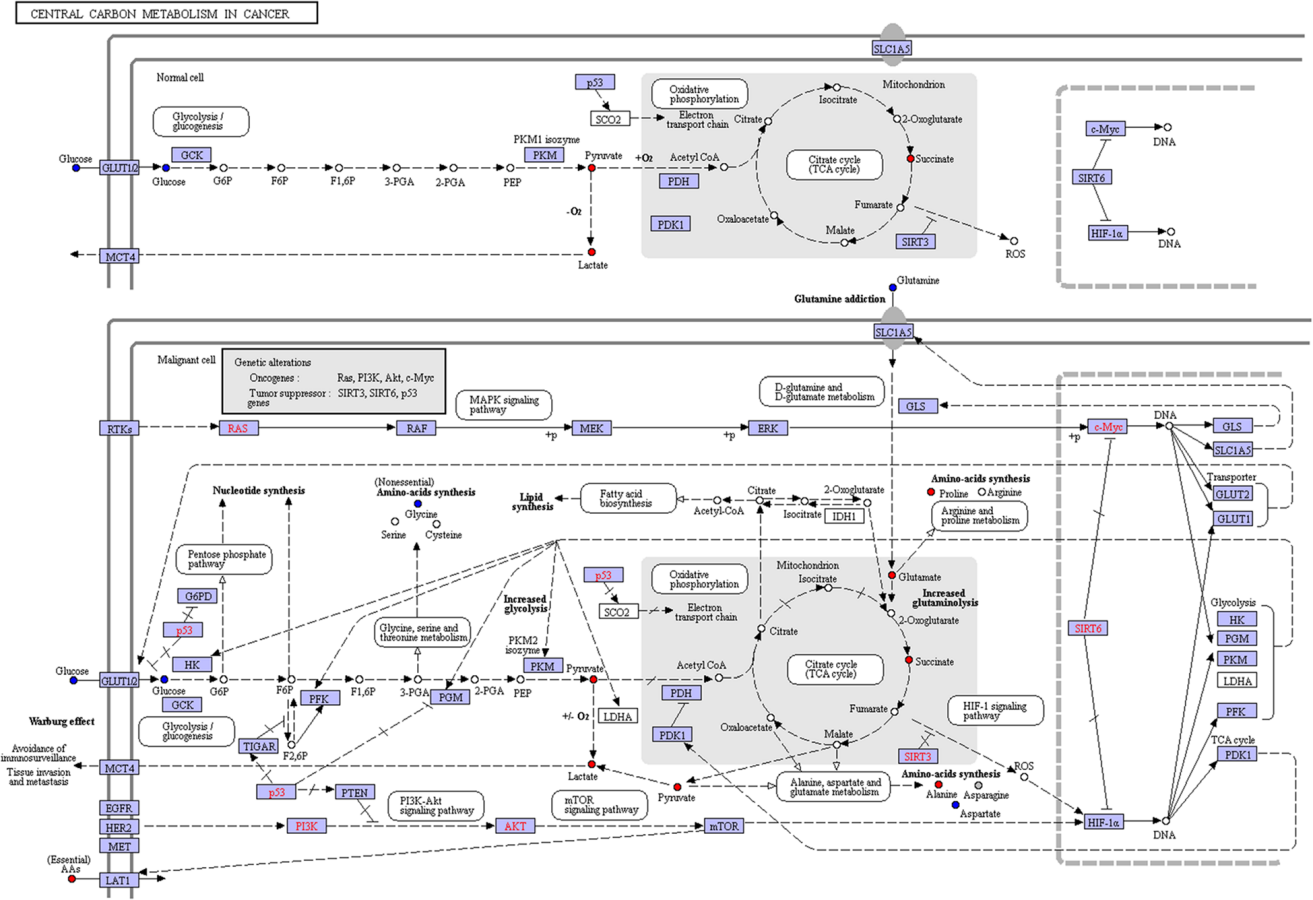
The metabolites and corresponding affected metabolic pathways generated by KEGG mapper. Nodes in red and blue denoting metabolites with increased and decreased concentrations in NSCLC group. Genetic alternations including oncogenes such as Ras, PI3K, Akt and c-Myc, and tumor suppressor genes such as SIRT3, SIRT6 and p53 were observed

The identified metabolites were mapped on the KEGG reference pathway diagram concerning central carbon metabolism in cancer (Fig. 7), where red and blue nodes represented increased and decreased metabolites in NSCLC patients, respectively. Some oncogene and tumor suppressor gene were found, such as the tumor suppressor gene of p53 [14] SIRT3 [15] and SIRT6 [16], and oncogene of Ras [17] PI3K [18], AKT [19] and c-Myc [20]. However, these genes were not found via any genetic exploration in this study, but are instead potentially linked to the measured downstream metabolic changes, which might reflect the potential treatment mechanism of MWA on NSCLC.

## Discussion

Lung cancer is a major contributor to cancer-related mortality and burden of disease. MWA can be used to provide potentially curative tumour ablation in patients who are not candidates for surgical resection [21]. The metabolic signature of serum from MWA treated NSCLC patients and underlying mechanisms has not been evaluated holistically. In this study, metabolic profile analysis was employed to extravagate the underlying mechanisms of MWA as a treatment of NSCLC. In serum of NSCLC patients, levels of lactate, alanine, glutamate, proline, glycoprotein, phenylalanine, tyrosine and tryptophan were increased, while levels of glucose, taurine, glutamine, glycine, phosphocreatine and threonine were decreased, concerning several possible mechanisms of hyperthermic cell killing such as rupture of plasma membrane, dysfunction of mitochondrial, DNA damage, and energy-based cell death.

### Destroy of membrane

Hyperthermia induced by MWA have been shown to change cell membrane integrity and fluidity, which were first considered to be the main cause of cell death [5, 22, 23]. Phosphocholine and choline, components of the plasma membranes, are often regarded as markers of the integrity of membranes. In our experiment, the slightly lowered levels of choline and O-phosphocholine in NSCLC group indicated an accelerated use of them for cancer cell proliferation. Compared with NSCLC, their levels in MWA group have no significant change. Ablation change the fluidity and permeability of cell membrane, thereby leading to concretion and cytolysis, subsequently causing intracellular metabolites shifts.

### Dysfunction of mitochondrial

Mitochondrial dysfunction has been well correlated with heat-induced injury [24]. Some ultrastructural changes was observed previously, such as dilatation of the mitochondria with rupture or loss of the cristae [24]. The level of taurine in NSCLC patients was decreased, which was in accordance with previous reports [11, 25–28]. As an antioxidant, taurine may be utilized as an antioxidant defense system against the oxidative stress involved in cancer process [29]. Ablation disrupted the mitochondrial membrane potential, resulting in the change in the redox status of cells [30], inducing cancer cell killing, thus leading to the reduced consumption of taurine.

### DNA injury

Glycine was found to be decreased, which was in accordance with the previous study [31]. As a simple, nonessential amino acid, glycine was involved in the production of DNA, phospholipids and collagen as well as the release of energy. Given that cancer cells reprogram their metabolisms comprehensively, the decreased level of glycine in NSCLC patients may be related with the accelerated DNA synthesis. The level of glycine was increased after hyperthermic ablation, indicating that hyperthermia treatment caused nucleotide damage. Previous research offers some support for this view, which indicated that hyperthermia could cause damage to DNA [30] and lead to inhibition of nucleolar RNA synthesis [24, 32, 33]. One possible mechanism of heat-induced DNA injury was the denaturation of some key replication enzymes, such as DNA polymerase α and β, which is responsible for DNA replication and repair synthesis, respectively [34]. Another potential mechanism could be attributed to the formation of endogenous reactive oxygen species (ROS) as a consequence of hyperthermic ablation [5], which subsequently results in DNA damage because DNA is particularly vulnerable to ROS-induced damage. As a result, imbalance of glycine related to DNA damage and repairs was found [35].

### Energy-based cell death

The level of lactate was increased in NSCLC patients, which was in accordance with the previous studies [11, 31, 36–38]. As previously reported, cancer cell metabolism involves primarily the conversion of glucose ultimately to lactate by an enzyme-catalyzed anaerobic fermentation rather than the oxidation of glucose ultimately to carbon dioxide and water as occurs with normal cells, known as the Warburg effect [39]. Recent study has proposed that lactate is also a tricarboxylic acid (TCA) cycle carbon source for NSCLC and sustain tumor metabolism in vivo [40].Therefore, the elevated levels of lactate found in the serum of NSCLC patients could be attributed to the enormous domestic for cell proliferation. Hyperthermia caused extensive damage of the ultrastructural of mitochondria, ruptured glycolysis and energy metabolism of cancerous cells, which blocking the generation of lactate as a fuel of TCA cycle.

Levels of serum alanine was elevated in NSCLC group, which was consistent with previous report [36]. Alanine is a glycogenic (glycogen-producing) amino acid that can be converted to pyruvate and tricarboxylic acid cycle intermediates, and then to glucose by gluconeogenesis, functioning as an energy source to meet the huge demand of energy consumed in various metabolic activities in tumor cells. Elevated levels of serum alanine may facilitate energy synthesis in the cells and provide enough energy for cell growth. Tumor cells also utilized glutamine as another energy supply [41], which was called glutaminolysis. The decreased level of glutamine has been reported previously [42, 43], which was possibly due to its consumption for energy supply. After MWA treatment, the elevated level of alanine and decreased level of glutamine were recovered to the status of normal control group. Destroy of mitochondria and coagulative necrosis of cancer cells caused by hyperthermia might be responsible for the reversion of the above metabolites which are related with energy metabolism.

However, the roles of the significant pathways mentioned above in relation to MWA treatment were only hypothesis, without any complementary techniques as a backing. The model without vigorous explaination and prediction ability limits us to draw any definitive conclusions, which may be attributed to the small sample size and the heterogeneity of patients. Future studies with a larger sample size are planned to more clearly characterize metabolic profiles of NSCLC patients.

## Conclusions

^1^H NMR-based metabolomics approach could effectively distinguish the metabolic profile of NSCLC patients with or without MWA treatment from that of healthy controls. The observed potential biomarkers may facilitate to diagnose NSCLC non-invasively. MWA method could partially reverse the disturbed metabolic profile towards the control group. In the future, more patients from different ethnicities should be enrolled to verify the precision and specificity of the potential biomarkers in the diagnosis of NSCLC and the efficacy evaluation of MWA in curing NSCLC in clinic.

## Additional file

Additional file 1: **Table S1.** Potential serum biomarkers identified by ^1^H NMR and their means and standard deviation values for each group. **Table S2.** Pathway analysis and the altered pathways using MetaboAnalyst. **Figure S1.** PCA scores plot of ^1^H NMR spectra from control, NSCLC and MWA groups. **Figure S2.** Scatter plots of the metabolites to show the interindividual variation. Gray circle: CTRL group, green circle: NSCLC group, red circle: MWA group. (DOCX 554 kb)

## Abbreviations

AKT: Protein kinase B
c-Myc: MYC proto-oncogene
DNA: deoxyribonucleic acid
HDL: high density lipoprotein
HMDB: Human metabolites database
KEGG: Kyoto Encyclopedia of Genes and Genomes
LDL: low-density lipoprotein
MMCD: Madison Metabolomics Consortium Database
MWA: microwave ablation
NMR: nuclear magnetic resonance
NSCLC: nonsmall-cell lung cancer
PCA: Principal Component Analysis
PI3K: phosphatidylinositol 3-kinase
PLS-DA: Partial least squares discriminant analysis
Ras: rat sarcoma
RNA: ribonucleic acid
ROS: Reactive Oxygen Species
SIRT3: sirtuin-3
SIRT6: Sirtuin 6
TCA: tricarboxylic acid
VLDL: very low density lipoprotein
