# Supplementary material for: Serum proton NMR metabolomics analysis of human lung cancer following microwave ablation
Source: Radiat Oncol. 2018 Mar 12;13:40. doi: 10.1186/s13014-018-0982-5 (PMC5848604; doi:10.1186/s13014-018-0982-5)
Supplement: Supplementary file 1 — Table S1. Potential serum biomarkers identified by 1H NMR and their means and standard deviation values for each group. Table S2. Pathway analysis and the altered pathways using MetaboAnalyst. Figure S1. PCA scores plot of 1H NMR spectra from control, NSCLC and MWA groups. Figure S2. Scatter plots of the metabolites to show the interindividual variation. Gray circle: CTRL group, green circle: NSCLC group, red circle: MWA group. (DOCX 554 kb) [file 13014_2018_982_MOESM1_ESM.docx]

**Table S1.** Potential serum biomarkers identified by ^1^H NMR and their means and standard deviation values for each group.

| No. | Metabolites | CTRL | | NSCLC | | MWA | |
| --- | --- | --- | --- | --- | --- | --- | --- |
|  |  | mean | sd | mean | sd | mean | sd |
| 1 | Isoleucine | 12.8016 | 6.3306 | 14.0079 | 7.3943 | 16.5127 | 8.6552 |
| 2 | Leucine | 135.011 | 24.434 | 146.592 | 30.327 | 135.613 | 22.204 |
| 3 | Valine | 85.1727 | 16.38 | 94.7967 | 26.587 | 110.485 | 41.973 |
| 4 | Isobutyrate | 9.93492 | 3.8934 | 10.5574 | 2.2263 | 11.5537 | 4.1184 |
| 5 | Ethanol | 32.2402 | 9.0132 | 32.4923 | 9.6546 | 36.1389 | 14.601 |
| 6 | 3-Hydroxybutyrate | 18.7356 | 4.5264 | 21.4573 | 7.186 | 23.3621 | 6.1635 |
| 7 | Lactate | 122.377 | 25.274 | 261.042 | 41.028 | 235.297 | 47.151 |
| 8 | Alanine | 85.6395 | 12.495 | 150.435 | 33.593 | 130.425 | 33.243 |
| 9 | Lysine | 134.535 | 26.127 | 150.887 | 44.532 | 166.31 | 35.057 |
| 10 | Acetate | 20.965 | 7.1729 | 24.6866 | 9.4657 | 26.654 | 8.9985 |
| 11 | Proline | 40.3796 | 13.05 | 58.6263 | 30.682 | 50.672 | 15.36 |
| 12 | Glycoprotein | 16.6478 | 3.8242 | 19.5663 | 5.8069 | 17.5118 | 4.9427 |
| 13 | Glutamate | 193.085 | 27.337 | 219.991 | 53.115 | 185.937 | 26.887 |
| 14 | Glutamine | 168.405 | 15.79 | 134.629 | 19.898 | 149.893 | 41.919 |
| 15 | Methionine | 37.5206 | 17.101 | 41.1007 | 14.926 | 45.7231 | 10.893 |
| 16 | Acetoacetate | 5.2513 | 2.2822 | 6.06014 | 1.7872 | 7.19417 | 3.2339 |
| 17 | Pyruvate | 1.92268 | 0.6371 | 2.28134 | 0.9716 | 1.94729 | 0.2914 |
| 18 | Succinate | 4.07294 | 0.907 | 4.93928 | 2.6665 | 4.00034 | 1.243 |
| 19 | Aspartate | 12.7334 | 3.4577 | 10.9872 | 4.7262 | 12.825 | 3.8099 |
| 20 | Asparagine | 13.2005 | 2.3649 | 11.6078 | 4.6058 | 13.4703 | 4.9418 |
| 21 | Phosphocreatine | 75.7106 | 12.518 | 61.5725 | 18.75 | 69.2707 | 18.233 |
| 22 | Choline | 45.8742 | 10.097 | 40.7131 | 15.911 | 43.6311 | 10.842 |
| 23 | O-Acetylcholine | 15.3601 | 4.7773 | 13.6403 | 2.8434 | 12.1552 | 4.558 |
| 24 | O-Phosphocholine | 4.52013 | 1.0656 | 4.02667 | 1.1554 | 3.44403 | 1.4955 |
| 25 | TMAO | 131.615 | 16.933 | 141.136 | 29.978 | 125.522 | 41.562 |
| 26 | Taurine | 193.422 | 36.197 | 146.969 | 30.135 | 203.064 | 51.607 |
| 27 | Glucose | 122.425 | 15.942 | 113.112 | 16.259 | 121.795 | 17.031 |
| 28 | Glycine | 69.3916 | 18.38 | 62.0742 | 11.86 | 69.5517 | 14.098 |
| 29 | Threonine | 31.1377 | 12.929 | 25.4009 | 5.6145 | 30.385 | 6.9323 |
| 30 | Glycerol | 5.35217 | 2.6452 | 6.65365 | 4.1246 | 5.42526 | 1.398 |
| 31 | dCTP | 40.8735 | 13.875 | 35.2433 | 12.064 | 30.1463 | 11.672 |
| 32 | Tyrosine | 93.7613 | 15.632 | 108.57 | 30.096 | 104.928 | 14.401 |
| 33 | Phenylalanine | 17.6305 | 2.4283 | 20.5477 | 6.5506 | 17.0715 | 8.536 |
| 34 | Tryptophan | 31.7164 | 3.7913 | 41.2776 | 13.962 | 36.5546 | 8.0185 |
| 35 | Histidine | 7.49834 | 2.7476 | 6.57139 | 1.8859 | 7.46763 | 2.4083 |
| 36 | Tyramine | 15.9936 | 3.6548 | 14.108 | 4.8675 | 13.6123 | 2.8169 |
| 37 | Formate | 2.70874 | 0.9548 | 2.3536 | 0.7943 | 2.64778 | 0.9217 |

**Table S2.** Pathway analysis and the altered pathways using MetaboAnalyst.

|  | Total | Expected | Hits | Raw p | #NAME? | Holm adjust | FDR | Impact |
| --- | --- | --- | --- | --- | --- | --- | --- | --- |
| Taurine and hypotaurine metabolism | 20 | 0.074782 | 2 | 0.002281 | 6.0831 | 0.17792 | 0.060826 | 0.36331 |
| D-Glutamine and D-glutamate metabolism | 11 | 0.04113 | 2 | 0.000672 | 7.3053 | 0.053755 | 0.053755 | 0.35294 |
| Alanine, aspartate and glutamate metabolism | 24 | 0.089738 | 2 | 0.003288 | 5.7176 | 0.25316 | 0.065755 | 0.26401 |
| Pyruvate metabolism | 32 | 0.11965 | 1 | 0.11366 | 2.1745 | 1 | 0.76125 | 0.13756 |
| Arginine and proline metabolism | 77 | 0.28791 | 2 | 0.031436 | 3.4598 | 1 | 0.35927 | 0.10231 |
| N-Glycan biosynthesis | 38 | 0.14209 | 1 | 0.13364 | 2.0126 | 1 | 0.76368 | 0.0206 |
| Starch and sucrose metabolism | 50 | 0.18695 | 1 | 0.17241 | 1.7579 | 1 | 0.81136 | 0.01703 |
| Primary bile acid biosynthesis | 47 | 0.17574 | 1 | 0.16287 | 1.8148 | 1 | 0.81136 | 0.00822 |
| Vitamin B6 metabolism | 32 | 0.11965 | 1 | 0.11366 | 2.1745 | 1 | 0.76125 | 0.00798 |
| Galactose metabolism | 41 | 0.1533 | 1 | 0.14348 | 1.9415 | 1 | 0.76525 | 0.00276 |
| Aminoacyl-tRNA biosynthesis | 75 | 0.28043 | 3 | 0.002133 | 6.1502 | 0.16851 | 0.060826 | 0 |
| Glycolysis or Gluconeogenesis | 31 | 0.11591 | 2 | 0.005464 | 5.2095 | 0.41528 | 0.087428 | 0 |
| Nitrogen metabolism | 39 | 0.14582 | 2 | 0.008573 | 4.7591 | 0.64297 | 0.11431 | 0 |
| Selenoamino acid metabolism | 22 | 0.08226 | 1 | 0.079443 | 2.5327 | 1 | 0.76125 | 0 |
| Pentose phosphate pathway | 32 | 0.11965 | 1 | 0.11366 | 2.1745 | 1 | 0.76125 | 0 |
| Methane metabolism | 34 | 0.12713 | 1 | 0.12037 | 2.1172 | 1 | 0.76125 | 0 |
| Propanoate metabolism | 35 | 0.13087 | 1 | 0.1237 | 2.0899 | 1 | 0.76125 | 0 |
| Cysteine and methionine metabolism | 56 | 0.20939 | 1 | 0.19121 | 1.6544 | 1 | 0.84984 | 0 |
| Pyrimidine metabolism | 60 | 0.22435 | 1 | 0.20354 | 1.5919 | 1 | 0.85699 | 0 |
| Amino sugar and nucleotide sugar metabolism | 88 | 0.32904 | 1 | 0.28522 | 1.2545 | 1 | 1 | 0 |
| Purine metabolism | 92 | 0.344 | 1 | 0.29625 | 1.2165 | 1 | 1 | 0 |


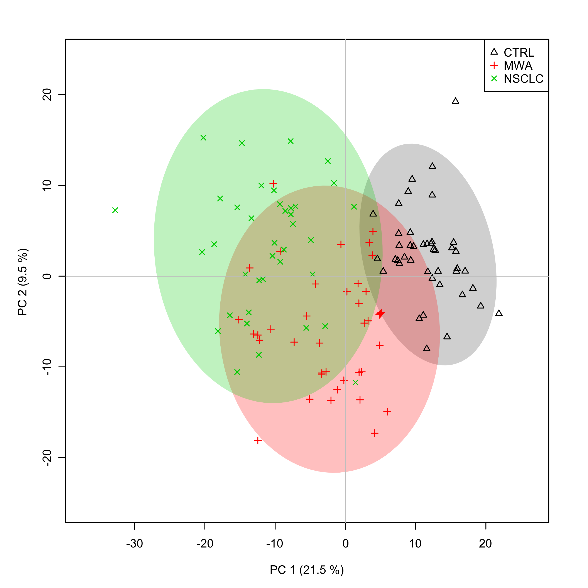


**Figure S1.** PCA scores plot of ^1^H NMR spectra from control, NSCLC and MWA groups.


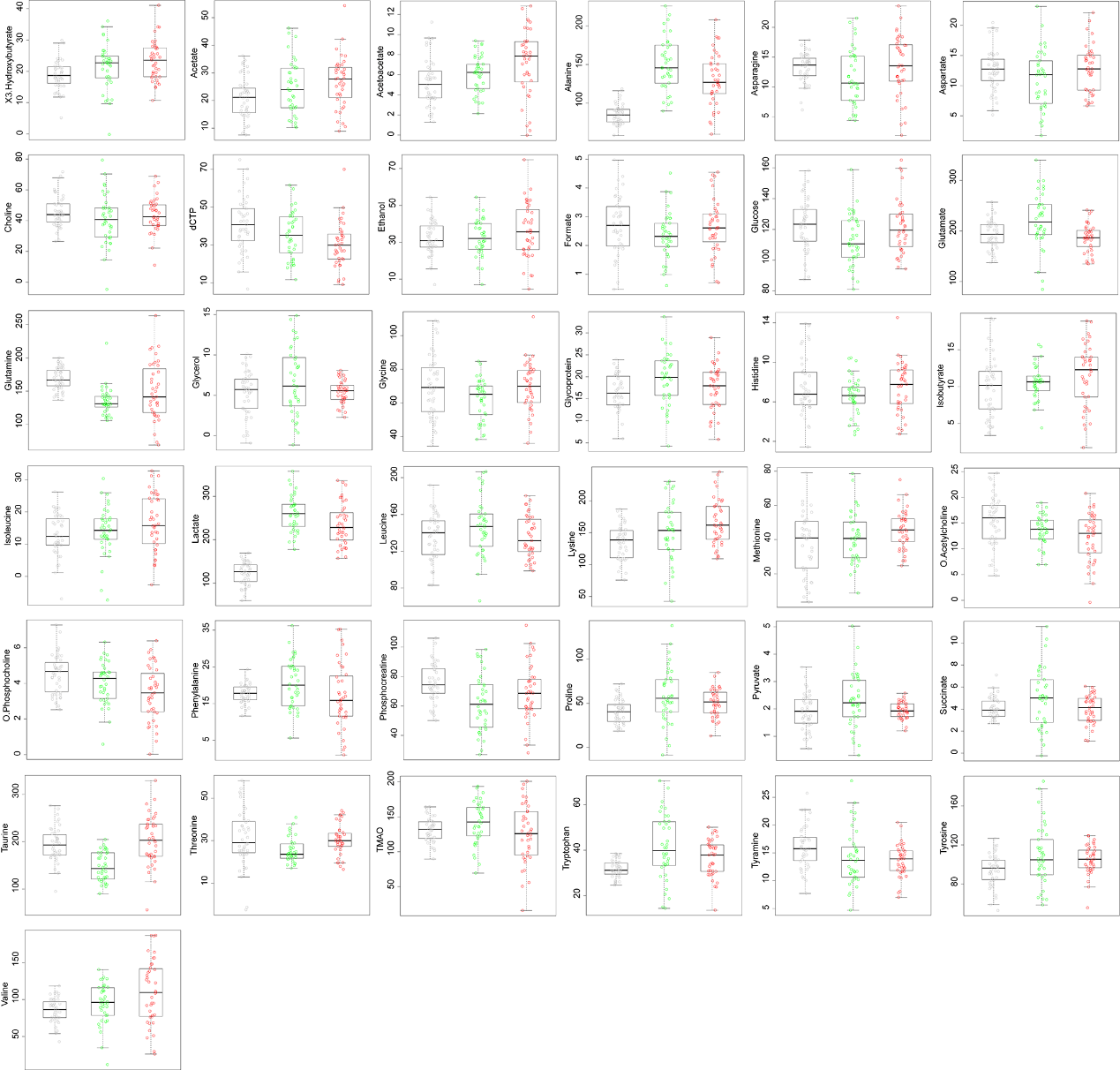


**Figure S2**. Scatter plots of the metabolites to show the interindividual variation. Gray circle: CTRL group, green circle: NSCLC group, red circle: MWA group.
